# Fasting upregulates the monocarboxylate transporter MCT1 at the rat blood-brain barrier through PPAR δ activation

**DOI:** 10.1186/s12987-024-00526-8

**Published:** 2024-04-08

**Authors:** Stéphanie Chasseigneaux, Véronique Cochois-Guégan, Lucas Lecorgne, Murielle Lochus, Sophie Nicolic, Corinne Blugeon, Laurent Jourdren, David Gomez-Zepeda, Stefan Tenzer, Sylvia Sanquer, Valérie Nivet-Antoine, Marie-Claude Menet, Jean-Louis Laplanche, Xavier Declèves, Salvatore Cisternino, Bruno Saubaméa

**Affiliations:** 1 Optimisation Thérapeutique en Neuropsychopharmacologie, Université Paris Cité, Inserm, 4 avenue de l’Observatoire, 75006 Paris, France; 2 Département de biologie, GenomiqueENS, Institut de Biologie de l’ENS (IBENS), École normale supérieure, CNRS, INSERM, Université PSL, 75005 Paris, France; 3 Helmholtz-Institute for Translational Oncology Mainz (HI-TRON Mainz), A Hemlholtz Institute of the DKFZ, Mainz, Germany; 4 German Cancer Research Center (DKFZ) Heidelberg, Division 191, 69120 Heidelberg, Germany; 5 Institute of Immunology, University Medical Center of the Johannes-Gutenberg University, Mainz, Germany; 6 Research Center for Immunotherapy (FZI), University Medical Center of the Johannes-Gutenberg University, Mainz, Germany; 7 AP.HP.Centre, Université Paris Cité, Paris, France; 8 AP-HP Biochimie générale, Hôpital Necker Enfants Malades, Université Paris Cité, Inserm, Innovations Thérapeutiques en Hémostase, Paris, France; 9 Institut de Chimie Physique, CNRS UMR8000, Université Paris-Saclay, 91400 Orsay, France

**Keywords:** Blood-brain barrier, Fasting, MCT1, PPAR δ, Endothelial cells

## Abstract

**Background:**

The blood-brain barrier (BBB) is pivotal for the maintenance of brain homeostasis and it strictly regulates the cerebral transport of a wide range of endogenous compounds and drugs. While fasting is increasingly recognized as a potential therapeutic intervention in neurology and psychiatry, its impact upon the BBB has not been studied. This study was designed to assess the global impact of fasting upon the repertoire of BBB transporters.

**Methods:**

We used a combination of in vivo and in vitro experiments to assess the response of the brain endothelium in male rats that were fed *ad libitum* or fasted for one to three days. Brain endothelial cells were acutely purified and transcriptionaly profiled using RNA-Seq. Isolated brain microvessels were used to assess the protein expression of selected BBB transporters through western blot. The molecular mechanisms involved in the adaptation to fasting were investigated in primary cultured rat brain endothelial cells. MCT1 activity was probed by in situ brain perfusion.

**Results:**

Fasting did not change the expression of the main drug efflux ATP-binding cassette transporters or P-glycoprotein activity at the BBB but modulated a restrictive set of solute carrier transporters. These included the ketone bodies transporter MCT1, which is pivotal for the brain adaptation to fasting. Our findings in vivo suggested that PPAR δ, a major lipid sensor, was selectively activated in brain endothelial cells in response to fasting. This was confirmed in vitro where pharmacological agonists and free fatty acids selectively activated PPAR δ, resulting in the upregulation of MCT1 expression. Moreover, dosing rats with a specific PPAR δ antagonist blocked the upregulation of MCT1 expression and activity induced by fasting.

**Conclusions:**

Altogether, our study shows that fasting affects a selected set of BBB transporters which does not include the main drug efflux transporters. Moreover, we describe a previously unknown selective adaptive response of the brain vasculature to fasting which involves PPAR δ and is responsible for the up-regulation of MCT1 expression and activity. Our study opens new perspectives for the metabolic manipulation of the BBB in the healthy or diseased brain.

**Supplementary Information:**

The online version contains supplementary material available at 10.1186/s12987-024-00526-8.

## Background

Fasting is increasingly recognized as a potential therapeutic intervention which might have a number of positive consequences in the healthy but also in the diseased individual [1]. In the field of neurology and psychiatry, fasting is currently scrutinized for possible beneficial effect in several conditions including stroke, multiple sclerosis, neurodegenerative diseases, cancer, major depression syndrome as long as metabolic syndrome and natural ageing which are major risk factors for neurological diseases [2–6]. Importantly, fasting and related metabolic interventions such as the ketogenic diet are already in clinical use for controlling seizures in drug-resistant epilepsy [7, 8]. Although fasting is accompanied with sustained hypoglycemia and despite the fact that the brain is often described as a glucose-dependent organ, it easily copes with this metabolic stress by switching its main energy nutrient from glucose to ketone bodies [9, 10]. In fact, fasting and calorie restriction are even thought to have neuroprotective actions by promoting the production of BDNF, dampening neuro-inflammatory processes, stimulating damaged organelles autophagy and enabling synaptic plasticity through activation of several signaling pathways such as the mTOR/AMPK, sirtuins and CREB axis [2, 4].

The blood-brain barrier (BBB) is a highly restrictive barrier between the blood and brain parenchyma supported by the endothelial cells of the cerebral vasculature. As compared to peripheral endothelial cells, cerebral endothelial cells (CECs) show a unique phenotype characterized by an elaborate complex of tight junctions, a very low rate of constitutive transcytosis and the expression of several ATP-binding cassettes (ABC) and Solute Carrier (SLC) transporters [11]. ABC transporters, in particular PGP (*Abcb1a*), BCRP (*Abcg2*) and MRP4 (*Abcc4*), are mostly known for restricting the brain distribution of a large panel of xenobiotics, including many drugs. By contrast, SLC transporters allow the regulated uptake of nutrients needed for brain function. Among those are GLUT1 (*Slc2a1*), which allows glucose to readily enter the brain and MCT1 (*Slc16a1*), which plays an essential role in shuttling lactate and ketone bodies into the brain, in particular when blood glucose availability is diminished. Very few studies have addressed the impact of fasting on brain endothelial cells. It has been reported that one day of fasting increases PGP activity at the mouse BBB [12]. Besides, the brain uptake of glucose and ketones, as measured by positron emission tomography, was increased following two days of fasting in the rat, while the endothelial expression of GLUT1 and MCT1 was unchanged [13]. However, the global response of the BBB to fasting, as well as the underlying molecular mechanisms, remain to be investigated.

Fasting drives a major metabolic switch involving in particular a sharp rise in the plasma concentration of free fatty acids (FFAs), which can be used as an alternate energy source or converted to ketone bodies in the liver [1]. This rise in FFAs activates a major lipid sensing system composed of nuclear receptors of the peroxisome proliferator-activated receptor (PPAR) family. PPARs are activated by a number of saturated and unsaturated FFAs as well as lipid-derived compounds (such as arachidonic acid derivatives or endocannabinoids) [14] and are pivotal for the adaptation to fasting in several organs including the liver and muscle [15, 16]. This family comprises three isoforms (α, δ, γ encoded respectively by *Ppara*, *Ppard*, *Pparg*) which share significant overlap in their ligands, DNA binding sequence and target genes [17]. Nevertheless, they are not always functionally redundant and their activation can have distinct functional consequences depending upon tissue-specific features such as their relative expression and the presence of specific interacting partners [18]. For example, PPAR γ promotes adipogenesis and lipid storage in the adipose tissue, while PPAR α and PPAR δ enhance fatty acids oxidation, respectively, in the liver and muscle [16, 19]. However, the effects of fasting on CECs and on the expression and function of PPARs at the BBB have not been studied yet.

In the present study, we first assess the global impact of fasting upon the BBB by performing a whole transcriptomic profiling of freshly isolated CECs from fed vs. fasted rats. We show that fasting does not alter the expression of drug unidirectional efflux ABC transporters at the BBB but modulates the expression of a restrictive set of SLC transporters, mainly involved in the transport of nutrients, enzymatic cofactors and neurotransmitters, including MCT1. Using a combination of in vitro and in vivo approaches, we then demonstrate that fasting selectively activates PPAR δ in brain endothelial cells. Finally, we show that this activation is responsible for the upregulation of MCT1 expression and activity at the BBB. Altogether, we describe a new selective adaptive response that takes place in the cerebrovascular system during fasting.

## Methods

### Animals

Male Wistar-Han rats (Charles River Laboratories) weighing 300–350 g were used in this study. They were kept on a 12:12-hour dark:light cycle with *ad libitum* access to food and water. All experiments complied with the ethical rules of the European directive (2010/63/EU) for experimentation with laboratory animals and were approved by the ethics review committee of Paris Descartes University (approval no. 18–090 #20,159). For fasting experiments, food was withdrawn from the cage on day 0 at 8.30 am and the bedding was changed to eliminate pellet residues and limit coprophagy. Rats were then sacrificed 24 h, 48 h, or 72 h later at 8.30 am. Control animals were fed *ad libitum* and all animals had free access to water. Rats were weighed on day 0 and on the day of sacrifice. Glycemia and ketonemia were measured just before sacrifice on a drop of tail-tip blood using a Freestyle Optium Neo H (Abbott) with glucose and ketones strips. For in vivo inhibition of PPAR δ during fasting, rats were dosed with the specific inhibitor GSK3787 administered intra-peritoneally at 2 mg/kg every 12 h for 48 h, starting with food removal.

### Biochemical assays

Plasma total proteins were determined by Biuret’s method and plasma urea and free fatty acids were measured by enzymatic methods. These parameters were assessed on an Alinity Analyzer (Abbott).

### Chemicals

DMEM (cat. # 21885-025), DMEM-F12 (cat. # 10,565,018), HBSS (cat. # 14175-053), and HEPES (cat. # 15630-056) were from Gibco. Fetal Bovine Serum (FBS) (cat. # SV30160.03) was from HyClone. Liberase DL (cat. # 05401160001), Liberase DH (cat. # 5,401,089,001), Liberase TM (cat. # 05401119001), Bovine Serum Albumin (BSA) (cat. # A7906), Dextran (cat. # 31,390) and DNase I (cat. # DN25) were from Merck. PPAR agonists and antagonists were purchased from Merck (GW501516, GSK0660, GW6471, Fenofibrate, Rosiglitazone) or Euromedex (GW0742, GSK3787).

### Preparation of brain cell suspensions

Four rats were used for each isolation. Animals were deeply anesthetized by intra-peritoneal injection of 10 mg/kg diazepam (Bayer) and, ten minutes later, 100 mg/kg ketamine (Panpharma). Then, they were transcardially perfused with Buffer 1 (HBSS, 10 mM HEPES) containing 20 U/mL heparin (Panpharma) at 4 °C for 3 min to remove blood from the brain vasculature. All subsequent steps were done on ice except when indicated. The cortex was dissected, carefully cleared from adhering white matter and meninges and gently crushed in a Petri dish using a glass slide. Tissue pieces from two cortices were pooled in 30 mL of Buffer 1, rinsed twice by sedimentation in Buffer 1, then centrifuged (2 min, 600 g) and resuspended in 10 mL of DMEM containing 10 mM HEPES, 0.2 WU/mL Liberase DL, 0.2 WU/mL Liberase DH and 100 U/mL DNase I. Digestion was performed for 40 min at 37 °C with gentle mechanical trituration with a 10 mL pipette (at 10 min), then with a P1000 pipet tip (at 20 min) and finally with a rounded glass Pasteur pipette (at 30, 35, and 40 min), to obtain a homogenate with a creamy texture and almost no visible remaining piece. Digestion was stopped by adding 25 mL of DMEM + 10% FBS and the homogenate was centrifuged (10 min, 1,000 g). The supernatant was discarded and the pellet was resuspended in 25 mL of Buffer 1 + 18% BSA and centrifuged (15 min, 2,000 g). The floating compact myelin disk was eliminated and the pellet was resuspended in 50 mL of Buffer 1 + 1% BSA. This suspension was filtered sequentially on a 100 μm Cell Strainer (Corning) and a 10 μm nylon mesh (cat. # NY1004700, Millipore). Cells from the filtrate were pelleted and resuspended in Buffer 1 containing 300 µg/mL Liberase TM and 100 U/mL of DNase I for 30 min at room temperature. The digestion was stopped by adding 30 mL of DMEM + 10% FBS and undigested vessel fragments were removed by filtration on a 10 μm nylon mesh. Cells were finally pelleted, resuspended in MACS buffer (PBS with no Ca^2+^/Mg^2+^, 0.5% BSA, 2 mM EDTA) and counted.

### Magnetic-activated cell sorting of brain cortex endothelial cells

Anti-CD31-Phycoerythrin (PE) and anti-CD11b-Fluorescein Isothiocyanate (FITC) antibodies, anti-FITC and anti-PE magnetic microbeads, LS and LD columns were used following the recommendations of the manufacturer (Miltenyi Biotec). The whole procedure was performed at 4 °C and cells were washed in MACS buffer and recovered by centrifugation (5 min, 1,000 g) following each incubation step. Cerebral endothelial cells were isolated from brain cell suspensions by positive cell sorting. Cells were incubated sequentially in 50 µL of MACS buffer per million cells + 10% (v/v) anti-CD11b-FITC and 1% (v/v) anti-CD31-PE antibodies for 20 min. Cells were then incubated for 15 min in 90 µL of MACS buffer + 10 µL of anti-FITC microbeads (per 10 million cells) and passed over a LD column to retain CD11b + microglia. Unretained cells were labelled for 15 min in 80 µL of MACS buffer + 20 µL of anti-PE microbeads (per 10 million cells) and passed over a LS column. CD31 + endothelial cells were recovered in the retained fraction.

### Flow cytometry

Cells were stained with 7-AAD (4 mg/L for 20 min on ice) and analyzed on a BD Accuri™ C6 (BD Biosciences) flow cytometer. Debris and dead cells were excluded from the analysis by gating events on FSC/SSC and 7-AAD fluorescence, respectively.

### RNA-Seq

Following MACS, cells were immediately lysed and total RNA was extracted using the RNeasy® Micro Kit (Qiagen). Library preparation and Illumina sequencing were performed at the Ecole normale supérieure GenomiqueENS core facility (Paris, France). Messenger (polyA^+^) RNAs obtained in three independent experiments (biological replicates) were purified from 150 ng of total RNA using oligo(dT). All samples had RNA integrity numbers ranging from 9,1 to 9,9 as assessed by the Fragment Analyzer instrument (Agilent Technologies). Libraries were prepared using the strand-specific RNA-Seq library preparation TruSeq Stranded mRNA kit (Illumina). Libraries were multiplexed by twelve on a run. A 75 bp single-end read sequencing was performed on NextSeq 500 device (Illumina). A mean of 33 ± 1.7 million reads passing the Illumina quality filter was obtained for each of the 12 samples.

### RNA-Seq data analysis

The analyses were performed using the Eoulsan pipeline [20], including read filtering, mapping, alignment, filtering, and read quantification. Before mapping, poly N read tails were trimmed, reads ≤ 40 bases were removed, and reads with quality mean ≤ 30 were discarded. Reads were then aligned against the *Rattus norvegicus* genome from Ensembl version 91 using STAR (version 2.6.1b). Alignments from reads matching more than once on the reference genome were removed using Java version of samtools. To compute gene expression, *Rattus norvegicus* GTF genome annotation version 91 from Ensembl database was used. All overlapping regions between alignments and referenced exons were counted and aggregated by genes using HTSeq-count 0.5.3. The DESeq2 package (version 1.30.1) [21] was used for normalizing counts and estimating differential gene expression, which was expressed as LFC, defined as the Log_2_ of the fold change between fasting (F1, F2, F3) and *ad libitum* (AL) conditions, with corresponding adjusted p-values. The whole dataset is presented in Suppl. Table S1 and contain 13,049 genes with at least one read in each sample (after summation over technical replicates). The RNASeq gene expression data and raw fastq files are available on the GEO repository (www.ncbi.nlm.nih.gov/geo/) under accession number GSE241803.

### qRT-PCR

Reverse transcription was performed on 100 ng of total RNA using random primers (cat # N8080127) and the Superscript II Reverse Transcriptase (cat # 18,064,022) following the supplier’s recommendations (Thermo Fisher Scientific). For qPCR, 8 µL of cDNA diluted 1/20 was mixed with 10 µL of SYBR Green fluorescence detection solution (Thermo Fisher Scientific) and 1 µL of each primer (Eurogentec) (see Suppl. Table S5 for primers’ sequence). qPCR was performed in 384-wells plates on a CFX384 Touch Real-Time PCR Detection System (Bio-Rad Laboratories) using the following program: 2 min at 50 °C, 10 min at 95 °C, 40 cycles of amplification (15 s at 95 °C, 45 s at 60 °C). All primers had amplification efficiency close to 100% as checked by the analysis of standard dilution curves (slope close to -3.33) so that the relative expression of a gene *X* to the housekeeping gene *Tbp* could be calculated by 2^− ΔCt^ where ΔCt = Ct(*X*)-Ct(*Tbp*) and Ct is the threshold cycle value.

### Mechanical isolation of brain microvessels

Four rats were used for each isolation. Rats were anesthetized and transcardially perfused with heparinized Buffer 1, as described above. The whole procedure was performed at 4 °C. Cortices were cleared of meninges, recovered in 30 mL/two cortices of Buffer 1 and triturated in a Potter-Thomas homogenizer using 10 strokes at 400 rpm. The homogenate was centrifuged, washed once in Buffer 1, and resuspended in Buffer 1 + 17.5% Dextran. After centrifugation at 2,500 g for 15 min, the floating myelin disk was eliminated and the pellet was resuspended in Buffer 1 + 1% BSA. This suspension was filtered sequentially on a 100 μm Cell Strainer and a 10 μm nylon mesh and microvessels’ fragments were recovered from the 10 μm filter in Buffer 1 + 1% BSA. After rinsing in Buffer 1, microvessels were stored as a dry pellet at -80 °C until further processing.

### Western-blot

Brain microvessels were homogenized in RIPA buffer (50 mM Tris.HCl pH 7.4, 150 mM NaCl, 1% Triton X100, 0.5% Sodium Deoxycholate, 0.1% SDS, 1 mM EDTA) containing cOmplete™ Protease Inhibitor Cocktail (Merck). Protein concentration was measured using a commercial kit (microBCA, Thermo Fisher Scientific). Samples were mixed with 5X Laemmli buffer, incubated for 15 min at room temperature and loaded on SDS-PAGE gels. After migration, proteins were transferred onto a PVDF membrane. The membrane was blocked in Tris buffered saline (TBS) containing 5% fat-free milk for 1 h. For protein immunodetection, the membrane was incubated in primary antibodies overnight at 4 °C and in secondary HRP-conjugated antibodies for 1 h at RT. All antibodies (Suppl. Table S4) were diluted in blocking buffer. The membrane was thoroughly washed in TBS + 0.1% Tween 20 between all incubation steps. The signal was detected by chemiluminescence using the Pierce ECL Plus kit (Thermo Fisher Scientific) and the Azure 300 Imager (Azure Biosystems). When needed, membranes were stripped and reprobed using the same procedure as above. Quantification was performed in FiJi. Luminescence signal from β−actin (cultured cells and liver) or the endothelial marker CD31 (brain microvessels) was used to normalize the signal from the proteins of interest.

### In situ brain perfusion to assess PGP activity

The transport of [^3^H]-Digoxin into the brains of rats was measured using the in situ brain perfusion method [22]. Briefly, rats were deeply anesthesized as indicated above and their body temperature was maintained at 37–38 °C during surgery using a heating pad. The right external carotid and occipital arteries were ligated. Then, after ligation of the common carotid artery on the heart side, the right common carotid artery was catheterized with heparin-filled polyethylene tubing (0.76 mm i.d. × 1.22 mm o.d., Folioplast). The syringe containing the perfusion fluid was placed in an infusion pump (Harvard pump PHD 2000, Harvard APparatus) and connected to the catheter. The thorax of the animal was rapidly opened, the heart was cut and the perfusion was immediately started at a flow rate of 10 mL/min. The perfusion fluid was bicarbonate-buffered physiological saline (128 mM NaCl, 24 mM NaHCO_3_, 4.2 mM KCl, 2.4 mM NaH_2_PO_4_, 1.5 mM CaCl_2_, 0.9 mM MgCl_2_ and 9 mM D-glucose). The solution was gassed with 95% O_2_ and 5% CO_2_ to obtain a pH of 7.4 and warmed to 37 °C. [^3^H]-Digoxin (0.3 µCi/mL) and [^14^C]-Sucrose (0.1 µCi/mL), as a marker of physical integrity of the BBB, were added to the perfusate. Both radiotracers were purchased from Perkin Elmer. Control experiments were performed by adding 10 µM Elacridar (cat. # SML0486, Merck), a PGP/BCRP specific inhibitor. Ethanol and DMSO did not exceed, respectively, 0.08% and 0.02% in volume in the perfusate. Perfusion was terminated after 60 s by decapitating the animal. The brain was removed and dissected on ice. The right cerebral cortex was carefully cleaned from meninges and underlying white matter and weighed. Aliquots of the perfusion fluid were also collected and weighed to determine tracers’ concentrations in the perfusate. Samples were digested in 2 mL of Solvable (Perkin Elmer) at 50 °C and mixed with 9 mL of Ultima gold XR scintillation cocktail (Perkin Elmer). Both labels were counted simultaneously in a Tri-Carb liquid scintillation counter (Perkin Elmer).

BBB transport parameters were calculated as previously described [22]. The brain vascular volume $$ {V}_{vasc}$$ (µL/g) was estimated from the tissue distribution of [^3^H]-Sucrose, which diffuses very slowly across the BBB, using the following equation:

$$ {V}_{vasc}=\frac{{X}^{*}}{{C}_{perf}^{*}}$$

where $$ {X}^{*}$$ (dpm/g) and $$ {C}_{perf}^{*}$$ (dpm/µL) are the activity of [^14^C]-Sucrose in the right brain cortex and perfusion fluid, respectively. Transport across the BBB was expressed in terms of the apparent distribution volume ($$ {V}_{brain}$$) and uptake clearance ($$ {K}_{in}$$) using the following equations:

$$ {V}_{brain}=\frac{{X}_{total}- {V}_{vasc}{C}_{perf}}{{C}_{perf}}$$

$$ {K}_{in}=\frac{{V}_{brain}}{T}$$

where $$ {X}_{total}$$ (dpm/g) is the total (vascular + parenchymal) activity of [^3^H]-Digoxin measured in the right brain cortex, $$ {C}_{perf}$$ (dpm/µL) is the measured activity of [^3^H]-Digoxin in the perfusion fluid, and T is the perfusion time (sec).

**In situ brain perfusion to assess MCT1 activity**.

The transport of [^14^C]-L-Lactate into the brain of rats was measured using the in situ brain perfusion method as described above. Each animal was perfused with [^14^C]-L-Lactate (0.1 µCi/mL) in the presence of non-labeled 0.25 mM L-Lactate during 60 s before decapitation. Previous experiments with [^14^C]-Sucrose had shown that the brain vascular volume $$ {V}_{vasc}$$ was highly constant among all animals (10.69 ± 0.98 µL/g) so that we used this measured value to calculate the uptake clearance $$ {K}_{in}$$(µL/s/g) for [^14^C]-L-Lactate as detailed above.

### Primary culture of brain endothelial cells

Four to eight rats were used for each isolation. The same procedure as described above for the preparation of brain cell suspensions was used but with the following modifications. Cortices were submitted to a single digestion step in 10 mL of DMEM (per 2 cortices) containing 10 mM HEPES, 0.4 WU/mL Liberase DL and 100 U/mL DNase I. After myelin elimination and sequential filtering on the 100 μm cell strainer and 10 μm nylon mesh, cells were counted, resuspended in culture medium and plated in multiwell plates previously coated with rat tail Collagen (0.1 mg/mL for 2 h at 37 °C). The culture medium was DMEM/F12 + Glutamax containing 20% Plasma Derived Bovine Serum (PDBS) (cat. # 60-00-850, First Link), 1% Penicillin/Streptomycin (Gibco), 80 µg/mL heparin (PanPharma) and 5 ng/mL bFGF (cat. # F0291, Merck). Cells were cultured for 4 days in the presence of 4 µg/mL puromycin (cat. # 540,411, Calbiochem) to selectively kill non-endothelial cells. Culture medium was then changed and the cells allowed to grow until confluency (1–2 days). All experiments were conducted 5–6 days following brain dissection without any cell passaging, so as to limit phenotypic drift of cultured cells.

### Non-targeted proteomic analysis of primary cultured rat CECs using LC-MS/MS

Unless otherwise specified, all solvents (Ultra LC-MS grade) were purchased from Carl Roth and all chemicals were obtained from Merck. Carboxylate-modified paramagnetic beads (Sera-Mag SpeedBeads CAT # 45,152,105,050,250 and Sera-Mag SpeedBeads cat. # 65,152,105,050,250) were purchased from GE Healthcare. The trypsin used was Trypsin Gold Mass Spectrometry Grade (cat. # V5280) from Promega.

Primary cultured rat CECs (pc-CECs) were grown to subconfluency in 6-wells plates and treated as indicated in the Results (*n* = 3 wells/condition). At the end of the experiment, cells were detached from the plate using a 5 min incubation in Ca/Mg-free PBS, pelleted and the dry pellet was immediately frozen and stored at -80 °C until further analysis.

The cell pellets were carefully resuspended in lysis buffer (50 mM HEPES, 1% SDS) containing a Protease Inhibitor Complex (Roche). The samples were incubated for 5 min at 95 °C and then sonicated at 4 °C for 15 min using a Bioruptor (Diagenode). The cell lysates were submitted to tryptic digestion using a carboxylate-modified paramagnetic bead-based SP3 protocol [23] with modifications, as previously reported [24]. The peptides obtained were eventually recovered and acidified with 0.05% final formic acid (v/v) to be injected into LC-MS/MS.

LC-MS/MS analyses were conducted on a nanoElute LC system coupled to a timsTOF Pro 2 mass spectrometer (Bruker Daltonic) using data-independent acquisition with parallel accumulation– serial fragmentation mode (DIA-PASEF) [25, 26]. Reconstituted peptides were directly injected and separated on a reversed-phase C18 column (Aurora 25 cm x 75 μm 1.6 μm, IonOpticks). The column was heated to 50 °C. Mobile phase A was 0.1% formic acid (v/v) in water, and mobile phase B was 0.1% formic acid (v/v) in ACN. Peptides were loaded onto the column at 600 bar and separated using a linear gradient at 400 nL/min from 2 to 37% mobile phase B over 38 min. Finally, the column was rinsed at 95% B resulting in a total method time of 47 min. Eluted peptides were ionized in a CaptiveSpray Source, operated in positive ESI mode, with a capillary voltage of 1600 V and a dry temperature of 180 °C. The dual trapped ion mobility (TIMS) was configured to accumulate for 100 ms and then resolve the ions in a 100 ms TIMS ramp. MS profiling data (MS1) was acquired from 100 to 1700 m/z, covering an inversed mobility range (1/K_0_) from 0.6 V.s.cm^− 2^ to 1.6 V.s.cm^− 2^. Fragmentation spectra (MS2) were acquired in 36 isolation windows of 0.21 V.s.cm^− 2^ by 25 Th (1/K_0_ x m/z), with an overlap of 1 Th, from 304 to 1169 m/z. This resulted in acquisition cycles composed of 1 MS1 PASEF frame and 12 MS2 PASEF frames within 1.37 s. The collision energy was ramped linearly as a function of the inverse mobility from 59 eV at 1/K_0_ 1.3 V.s.cm^− 2^ to 20 eV at 1/K_0_ 0.85 V.s.cm^− 2^.

The chromatograms from LC-MS/MS experiments were analyzed using DIA-NN version 1.8.1 [27] by searching in the Uniprot *Rattus norvegicus* database supplemented with a list of contaminating proteins internal to the laboratory and allowing one missed tryptic cleavage. DIA-NN was configured with N-terminal methionine excision, oxidation of methionine residues as a variable modification and carbamidomethylation of cysteine residues as a fixed modification. Match Between Runs (MBR) was used and the precursors were filtered with a 1% FDR threshold. These results were statistically processed using the MSstats package version 4.9.5 [28]. Low power, resulting from small effects due to ratio compression and few replicates (here *n* = 3), is a common feature of quantitative proteomics experiments. Hence multiple testing correction is often too blunt to detect any significant difference between groups, yielding a high proportion of false-negative results [29]. Therefore, differential expression between conditions with p-values < 0.05 were considered statistically significant based on the linear mixed model, without correction for multiple testing. The set of proteins that were both upregulated by GW0742 relative to control and downregulated by GW0742 + GSK0660 relative to GW0742 was analyzed using Kyoto Encyclopedia of Genes and Genomes to identify the corresponding enriched pathways.

### Statistical analysis

All data are presented as Mean ± SD. Statistical analysis was performed on GraphPad Prism 9.3.1. The Shapiro-Wilk test and the Brown-Forsythe test were used, respectively, to assess the normality and sphericity (equal variance across all groups) of the data. Normally distributed data were analyzed using One-way ordinary ANOVA followed by Holm-Sidak’s multiple comparisons test, when sphericity was satisfied, or Brown-Forsythe and Welch ANOVA followed by Dunnett’s multiple comparisons test, when sphericity was not satisfied. Data that were not normally distributed were analyzed using the non-parametric Kruskal-Wallis test followed by Dunn’s multiple comparisons test. For qRT-PCR, ΔCt = Ct (*gene of interest*)– Ct(*housekeeping gene*) values were used for testing. Data from in vitro experiments on primary cultured cells were obtained from 3 or 4 independent cultures and analyzed with pairing based upon the culture batch. * *p* < 0.05, ** *p* < 0.01, *** *p* < 0.001, **** *p* < 0.0001.

## Results

### Fasting triggers a metabolic switch in the rats

**Fig. 1:**
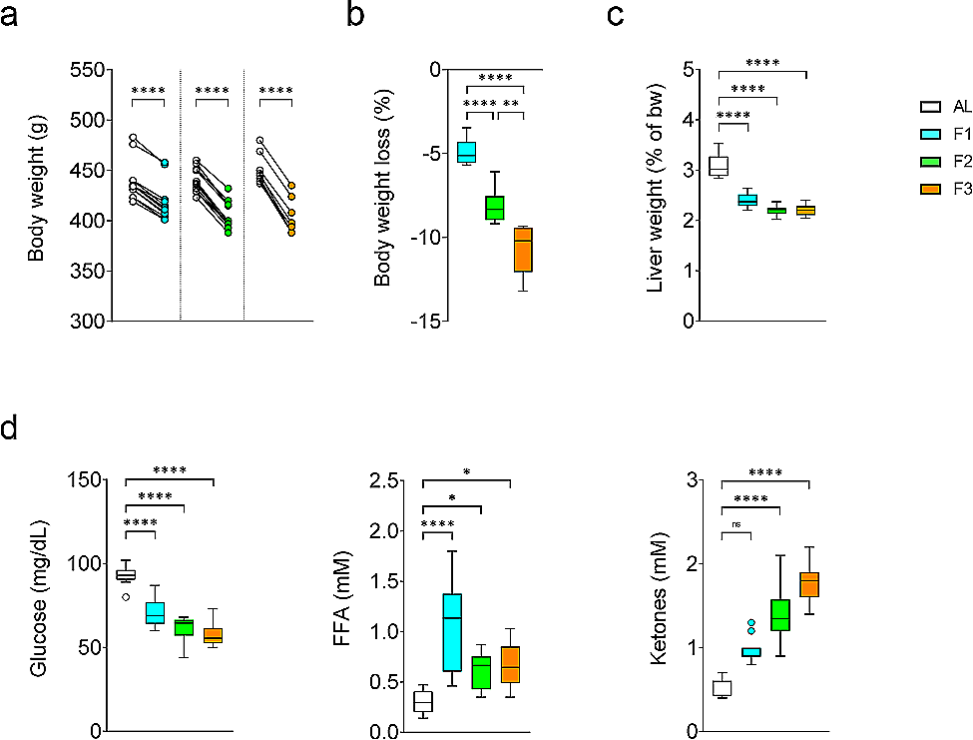
Metabolic effect of fasting. Rats were fed *ad libitum* (AL) or fasted for one day (F1), two days (F2), or three days (F3). (**a**) Body weight. Two-tailed paired t-test. (**b**) Change in body weight (%). One-way ANOVA followed by Holm-Sidak’s multiple comparisons test. (**c**) Liver relative weight (% of body weight, bw). Brown-Forsythe and Welch ANOVA followed by Dunnett’s multiple comparisons test. (**d**) Glucose, Free Fatty Acids (FFA) and Ketones plasma concentration. One-way ANOVA followed by Holm-Sidak’s multiple comparisons test for Glucose and FFA. Kruskal-Wallis followed by Dunn’s multiple comparisons test for Ketones. (**b**,**c**,**d**) Tukey boxplot (*n* = 12 rats per group)

Rats were fasted for one (F1), two (F2), or three (F3) days or fed *ad libitum* (AL). They lost an average of 4.8%, 8.1%, and 10.7% of their initial body weight following one, two, and three days of fasting respectively (Fig. 1a,b) while their liver’s relative weight was markedly decreased from fasting day one (Fig. 1c). As shown in Fig. 1d, the glycemia decreased, while the ketonemia and plasma concentration of FFAs increased during fasting, confirming that the expected metabolic switch characteristic of fasting occurred in our experimental animals. The plasma total protein concentration was unchanged in F1 and F2 but decreased by 18% in F3 rats, while the uremia was increased by 31% in F1 but returned to normal in F2 and F3 rats (Suppl. Fig. S1a). Moreover, the relative weight of the heart and several skeletal muscles was not decreased by fasting indicating the absence of significant protein catabolism in muscles (Suppl. Fig. S1b).

Altogether, these data indicate that one to three days of fasting in the adult male Wistar-Han rat is a safe procedure triggering a physiological metabolic switch as observed in the human.

### Fasting changes the transcriptomic profile of CEC

**Fig. 2:**
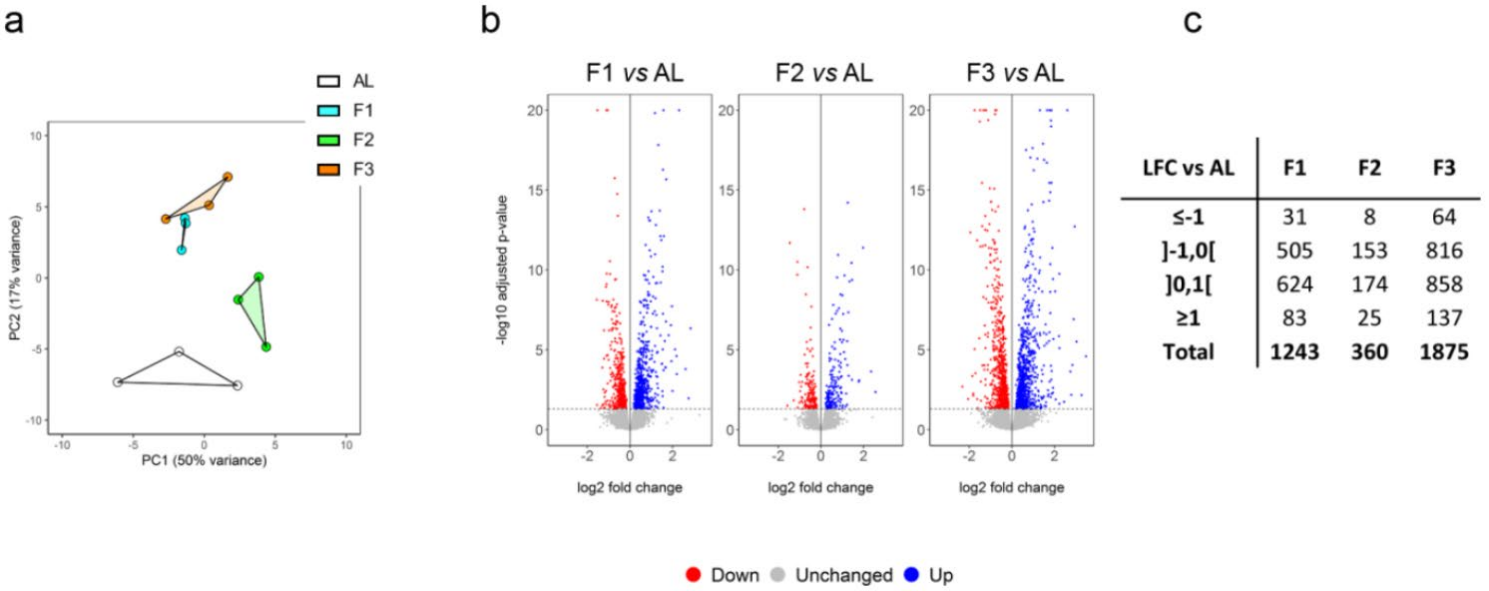
RNA-Seq analysis of MACS-purified brain endothelial cells. Rats were fed *ad libitum* (AL) or fasted for one day (F1), two days (F2), or three days (F3). (**a**) Principal Components Analysis. (**b**) Volcano plots showing differentially expressed genes (adjusted p-value ≤ 0.05) in F1, F2, or F3 vs. AL. (**c**) Number of differentially expressed genes in each group as a function of log2 fold change (LFC) vs. AL

We have previously described a procedure for the isolation of pericytes and vascular smooth muscle cells from the rat brain using magnetic-assisted cell sorting (MACS) [30]. For the present study, this procedure was slightly modified to isolate CECs from the cortex of fed and fasted rats (Suppl. Fig. S2a). The purity of the isolated CECs was close to 99% as assessed by CD31 (an endothelial-specific marker) staining and flow cytometry analysis (Suppl. Fig. S2b). We then performed a whole transcriptome analysis using RNA-Seq (Suppl. Table S1). Cell purity was confirmed by the undetectable or very low expression of glial, neuronal and mural cell-specific transcripts [31] in the resulting dataset (Suppl. Fig. S2c). Principal Components Analysis clearly distinguished the gene expression profile of F1 and F3 rats as compared to AL rats, while the overall difference in whole genome expression was more limited between the F2 and AL groups (Fig. 2a). This suggests that the global transcriptional response of CECs to fasting is biphasic with an initial early response at day one, followed by a transient partial return to the basal state at day two and a sustained response at day three. Accordingly, we found 1243, 360, and 1875 differentially expressed genes after, respectively, one, two, and three days of fasting relative to *ad libitum* feeding (Fig. 2b-c and Suppl. Table S1). Of note, at the level of individual genes, several kinetic patterns could be evidenced. For example the gene expression of *Angptl4* and *Pdk4* was upregulated at day one and day three but unchanged at day two (V-shaped profile), while the gene expression of *Slc16a1* was upregulated at all time points but maximum at day two (Λ-shaped profile).

### Fasting does not significantly impact the expression and function of the major drug efflux transporters at the BBB

**Fig. 3:**
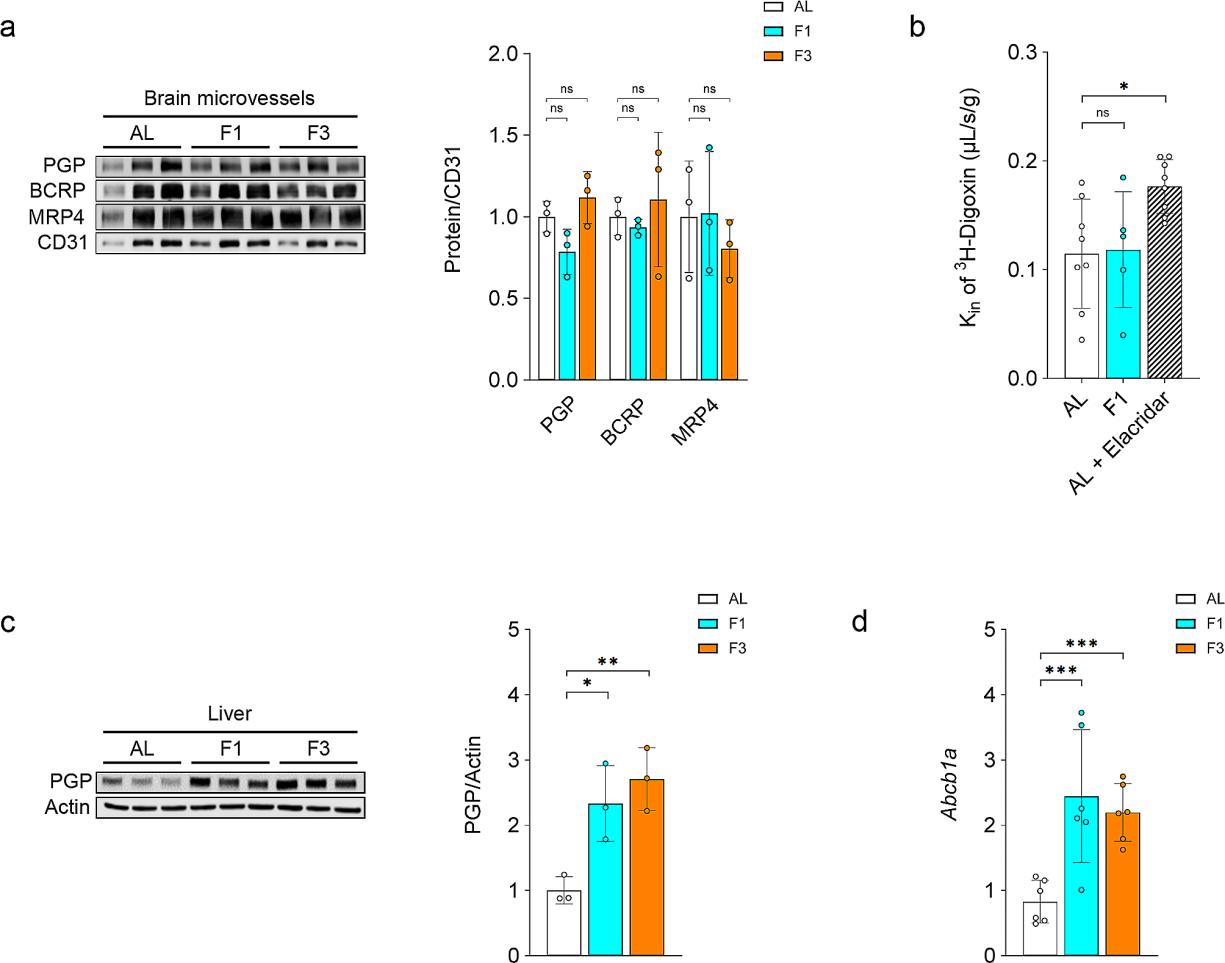
Effect of fasting on the expression of ABC transporters in brain microvessels and liver. Rats were fed *ad libitum* (AL) or fasted for one day (F1) or three days (F3). (**a**) Western-blot analysis of the expression of PGP, BCRP, and MRP4 in brain microvessels. For quantitative comparison, signals were normalized upon the signal from the endothelial-specific marker CD31. (**b**) Uptake clearance (K_in_) of the PGP specific substrate [^3^H]-Digoxin as measured by in situ brain perfusion in AL rats, F1 rats and AL rats in the presence of the PGP-specific inhibitor Elacridar as a positive control. (**c**) Western-blot analysis of the expression of PGP in the liver. For quantitative comparison, the signal was normalized upon the signal from Actin. (**d**) qRT-PCR analysis of the relative gene expression of *Abcb1a*. (**a-d**) Mean ± SD. One-way ANOVA with Holm-Sidak’s multiple comparisons test

The expression of several ABC-transporters is a hallmark of the brain endothelium, which supports the BBB. Among the 38 genes encoding ABC transporters that were detected in the transcriptome of the CECs, only four (*Abca3*, *Abca8a*, *Abcb9*, *Abcd1*) were differentially expressed in F1, F2, and/or F3 rats as compared to AL animals (Suppl. Table S2). Importantly the mRNA expression of *Abcb1a*/PGP, *Abcg2*/BCRP, and *Abcc4*/MRP4, encoding the three major drug efflux transporters at the BBB, did not change upon fasting. This was confirmed at the protein level using western-blot analysis of isolated brain cortex microvessels (Fig. 3a). Moreover, assessment of PGP activity in those animals, using the highly sensitive method of in situ brain perfusion, evidenced no change in the uptake clearance (K_in_) of the PGP substrate digoxin, while co-perfusion of the specific PGP inhibitor Elacridar increased the K_in_ value by 54% (Fig. 3b). By contrast, and as previously reported in the mouse [32], the expression of PGP was robustly increased at the protein level (Fig. 3c) and mRNA level (Fig. 3d) in the liver of F1 and F3 rats, relative to AL animals.

Altogether, these results show that fasting does not change the expression of the main drug efflux transporters or PGP activity at the BBB.

### Fasting changes the gene expression of a specific subset of Solute Carrier (SLC) transporters at the BBB

Among the 228 genes encoding SLC transporters detected in the transcriptome of the CEC, 49 transcripts were differentially expressed in F1, F2, and/or F3 rats as compared to AL animals (Suppl. Table S2). These transporters included in particular transporters of energy substrates such as glucose (*Slc2a1*/GLUT1), lactate and ketone bodies (*Slc16a1*/MCT1) and glutamine (*Slc38a3*/SNAT3), transporters of precursors of essential cofactors such as thiamine (*Slc19a3*/THTR2) and riboflavine (*Slc52a2*/RFT3) and transporters of neurotransmitters such as glutamate (*Slc1a1*/EAAT3) and taurine (*Slc6a6*/TAUT). In particular, *Slc16a1* expression significantly increased after one (Log_2_FC = 0.56), two (Log_2_FC = 1.26) and three (Log_2_FC = 0.79) days of fasting.

Given the essential role of MCT1 to sustain brain energy supply during fasting, we decided to further investigate the molecular mechanism supporting its induction.

### In vivo fasting and in vitro exposure to fatty acids activate PPAR δ in rat brain endothelial cells

**Fig. 4:**
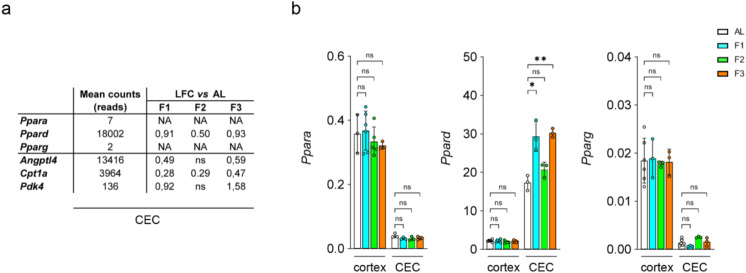
Effect of fasting on PPAR isoforms and target genes in CECs and whole cortex. Rats were fed *ad libitum* (AL) or fasted for one day (F1), two days (F2), or three days (F3). (**a**) Mean counts and Log2 fold change (LFC) relative to AL rats for selected transcripts (ns: not significantly different from AL, NA: LFC cannot be calculated because of too low expression). These figures were extracted from the whole RNA-Seq dataset presented in Suppl. Table S1. (**b**) Measurement of the abundance of *Ppara*, *Ppard* and *Pparg* transcripts in MACS-isolated rat CECs and whole cortex by qRT-PCR. Mean ± SD. One-way ANOVA with Holm-Sidak’s multiple comparisons test

*Slc16a1* has been previously reported as a PPAR α target gene in the liver [33]. Moreover, the rise in circulating FFAs and the subsequent activation of the PPAR family of transcription factors are a hallmark of fasting [15, 34]. Our RNA-Seq analysis revealed that *Ppard* (18,002 reads) is by far the most highly expressed isoform in freshly isolated CECs as compared to *Ppara* (7 reads) and *Pparg* (2 reads) (Fig. 4a). This was confirmed by qRT-PCR which additionally showed that *Ppard* mRNA expression was about eight times higher in CECs than in the whole cortex, indicating a selective enrichment in brain ECs as compared to neuronal and glial cells (Fig. 4b). Lastly, the mRNA expression of *Ppard* and several typical PPAR target genes including *Cpt1a*, *Angptl4* and *Pdk4* was increased by fasting in CECs, while *Ppara* and *Pparg* transcripts abundance remained unaffected (Fig. 4a-b), suggesting that PPAR δ is selectively activated in CECs during fasting.

**Fig. 5:**
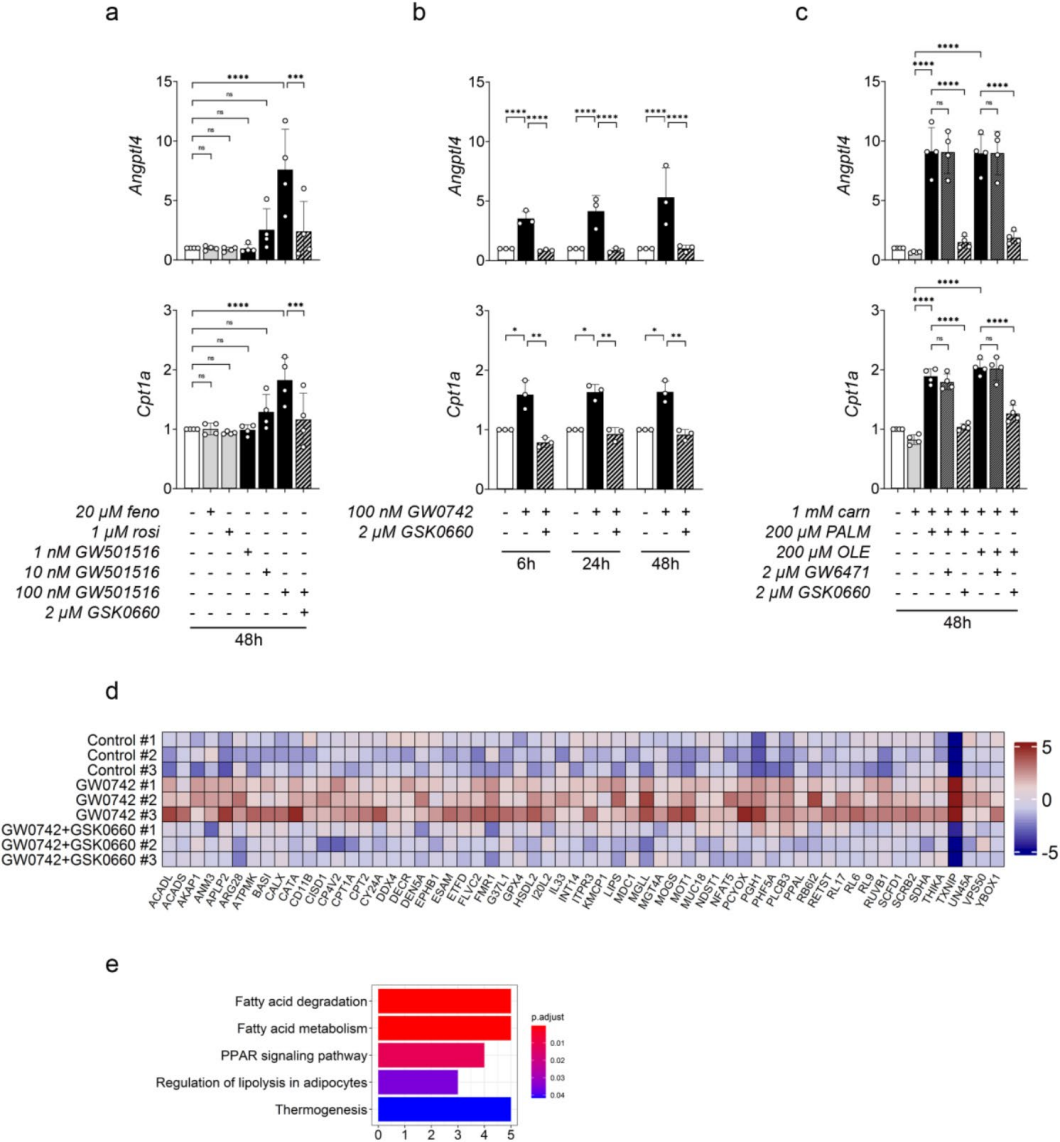
Effect of PPAR activation in primary cultured rat CECs. (**a-c**) qRT-PCR analysis of *Angptl4* and *Cpt1a* gene expression. (**a,b**) Cells were treated as indicated with synthetic agonists selective for PPAR α (fenofibrate, feno), PPAR δ (GW501516, GW0742), or PPAR γ (rosiglitazone, rosi) and synthetic antagonists selective for PPAR α (GW6471) or PPAR δ (GSK0660). (**c**) Cells were treated as indicated with palmitate (PALM) or oleate (OLE) in the presence of L-carnitine (carn). (**a-c**) Data are presented as fold increase to the control. Mean ± SD. One-way ANOVA with Holm-Sidak’s multiple comparisons test. (**d**) Non-targeted quantitative proteomics of CECs treated for 48 h with 100 nM GW0742 ± 2 µM GSK0660 or left untreated (Control) (*n* = 3). Heatmap of the standard deviations from mean protein intensities for the 60 proteins simultaneously upregulated in GW0742 vs. Control and downregulated in GW0742 + GSK0660 vs. GW0742 (p value ≤ 0.05). Note that MOT1 is the official name of MCT1 in the UniProt database. (**e**) KEGG enriched pathways in the proteins shown in d. (**a**-**e**) Culture medium contained 0.38% BSA in all conditions including control

To directly assess the functionality of PPAR δ in the brain endothelium, we first treated primary cultured rat CECs (pc-CECs) with selective pharmacological agonists of PPAR α (fenofibrate), PPAR δ (GW501516), and PPAR γ (rosiglitazone) and measured the expression of *Angptl4* and *Cpt1a* 48 h later. As shown in Fig. 5a, the gene expression of *Angptl4* and *Cpt1a* was dose-dependently increased by GW501516 but neither by 20 µM fenofibrate nor 1 µM rosiglitazone. Kinetic analysis using GW0742, another PPAR δ selective agonist, showed that this induction was already maximal after six hours of exposure (Fig. 5b). Moreover, the effects of GW501516 and GW0742 were blocked by the specific PPAR δ antagonist GSK0660.

FFAs are natural dietary PPAR ligands whose concentration rises in the blood during fasting. Their physiological plasma concentration in both human and rodents is in the range of a few hundred µM. Treatment of pc-CECs with 200 µM palmitate or oleate increased the expression of *Angptl4* and *Cpt1a*, an effect almost completely reversed by GSK0660 but not by the specific PPAR α antagonist GW6471 (Fig. 5c).

To further confirm that the PPAR δ pathway is active in brain endothelial cells, we exposed pc-CECs to GW0742 in the presence or absence of GSK0660 for 48 h and analyzed the cell lysate using non-targeted quantitative proteomics (*n* = 3). Among the 3367 proteins detected, 60 proteins, including CPT1A and MCT1 (annotated as MOT1 in Uniprot), were both upregulated by GW0742 relative to control and downregulated by GSK0660 relative to GW0742 and therefore considered as specifically upregulated by PPAR δ (Fig. 5d and Suppl. Table S3). Interestingly, KEGG analysis of these 60 proteins identified “PPAR signaling pathways”, “Fatty acid degradation” and “Fatty acid metabolism” as significantly enriched pathways (Fig. 5e). Moreover, several enzymes involved in lipid catabolism such as ACADL, ACADS, CPT1A, CPT2, MGLL or LIPS were found upregulated both at the protein level upon GW0742 exposure in vitro (Fig. 5d) and at the transcript level upon fasting in vivo (Suppl. Table S1).

Altogether, the results obtained in freshly isolated and primary cultured CECs show that fasting triggers the selective activation of PPAR δ in rat brain endothelial cells, possibly due to the rise in circulating FFAs.

### PPAR δ activation increases *Slc16a1*/MCT1 expression in rat brain endothelial cells in vitro

**Fig. 6:**
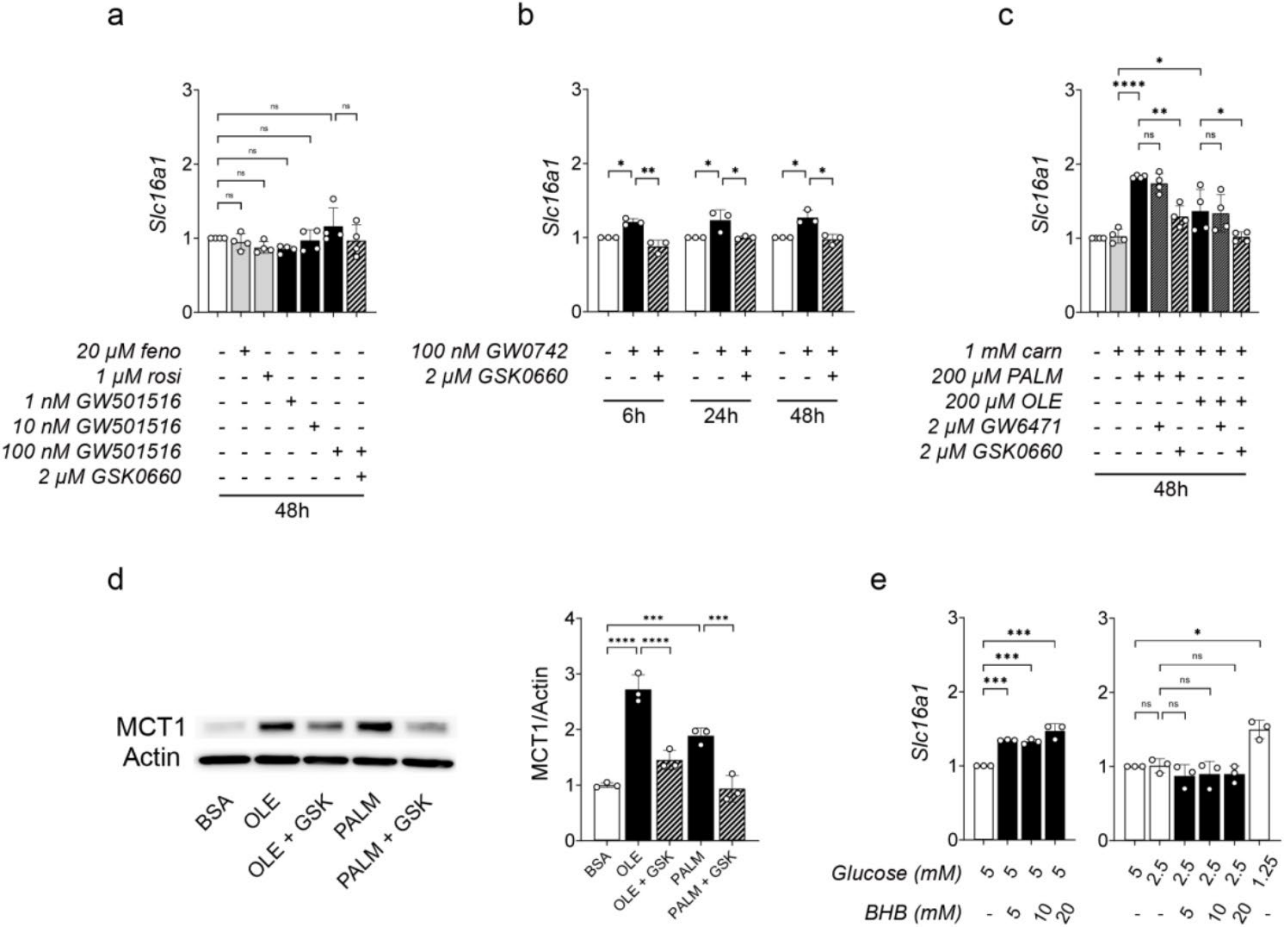
Effect of PPAR activation upon the expression of *Slc16a1*/MCT1 in primary cultured rat CECs. (**a-c**) qRT-PCR analysis of the gene expression of *Slc16a1*. (**a,b**) Cells were treated as indicated with synthetic agonists selective for PPAR α (fenofibrate, feno), PPAR δ (GW501516, GW0742), or PPAR γ (rosiglitazone, rosi) and synthetic antagonists selective for PPAR α (GW6471) or PPAR δ (GSK0660). (**c**) Cells were treated as indicated with palmitate (PALM) or oleate (OLE) in the presence of L-carnitine (carn). (**d**) Western-blot analysis of the relative expression of MCT1 in cells treated with 400 µM Oleate or 400 µM Palmitate ± 2 µM GSK0660 (GSK) for 48 h. For quantitative comparison, the signal was normalized upon the signal from Actin. (**e**) qRT-PCR analysis of CECs cultured with various concentrations of Glucose and β-hydroxybutyrate (BHB) for 48 h. The culture medium contained 0.38% BSA in all conditions including control (except in **e**). (**a-e**) Mean ± SD (fold increase to the control). One-way ANOVA with Holm-Sidak’s multiple comparisons test

We then asked whether the expression of *Slc16a1*/MCT1 expression was under the control of PPAR δ in pc-CECs. Fenofibrate, rosiglitazone, and GW501516 were unable to increase the gene expression of *Slc16a1* (Fig. 6a). However, this gene was modestly (+ 21–27%) but significantly upregulated by GW0742, an effect abrogated by the specific PPAR δ antagonist GSK0660 (Fig. 6b). Moreover, stimulation of pc-CECs with palmitate or oleate at physiological concentrations (200–400µM) significantly increased the expression of *Slc16a1*/MCT1 both at the gene level (Fig. 6c) and the protein level (Fig. 6d). According to the above results, *Slc16a1* induction was unsensitive to the PPAR α inhibitor GW6471, but reversed by the PPAR δ antagonist GSK0660 (Fig. 6c).

Besides a rise in plasma FFAs, fasting is accompanied by a drop in glycemia and a rise in ketonemia. In our rats, the mean glycemia was 3.8 mM and 3.3 mM, respectively, after one and three days of fasting, while the ketonemia reached a maximum of about 2 mM after three days of fasting (Fig. 1c). In vitro, the gene expression of *Slc16a1* was not increased when the glucose concentration of the medium was changed from 5 mM (mimicking normoglycemia) to 2.5 mM but increased when glucose concentration was further decreased to 1.25 mM (Fig. 6e, right graph, open bars). When pc-CECs were cultured for 48 h in the presence of 5 mM glucose (normoglycemia) plus 5, 10, or 20 mM BHB, the gene expression of *Slc16a1* was slightly increased (Fig. 6e, left graph, closed bars). However, in the presence of 2.5 mM glucose, which better mimicks the hypoglycemia which occurs during fasting in vivo, 5 to 20 mM of BHB did not change the gene expression of *Slc16a1* (Fig. 6e, right graph, closed bars).

Altogether, these results show that the selective activation of PPAR δ by FFAs, but neither low glucose, nor high ketones, at least in conditions mimicking a physiological fasting, are able to increase the expression of *Slc16a1*/MCT1 in rat brain endothelial cells in vitro.

### Fasting increases MCT1 protein expression at the BBB via PPAR δ in vivo

**Fig. 7:**
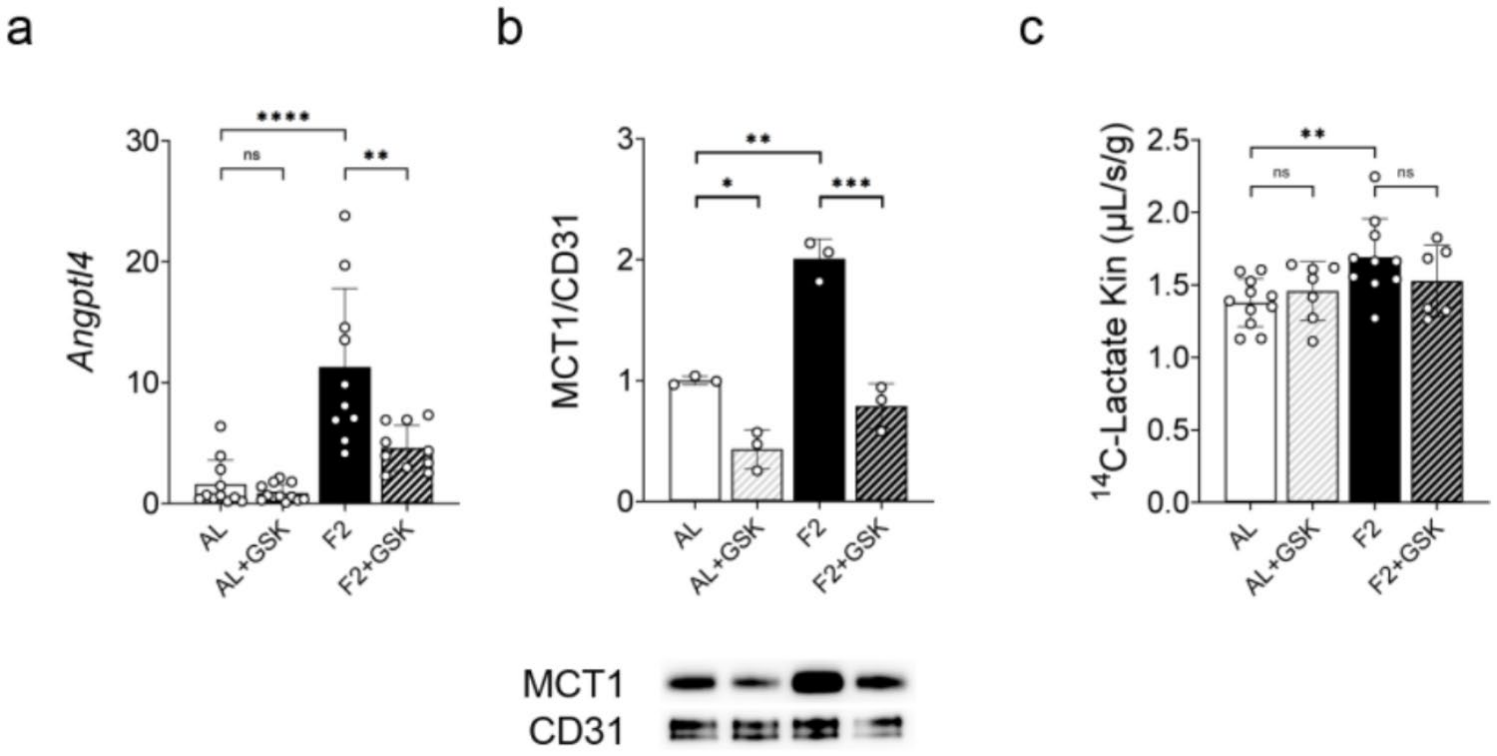
Fasting increases MCT1 expression and activity in vivo. Rats were fasted for two days (F2) or fed *ad libitum* (AL) and dosed concomitantly with GSK3787 (GSK) (2 mg/kg i.p. every 12 h) or vehicle. (**a**) qRT-PCR analysis of *Angptl4* relative gene expression in soleus muscle. Mean ± SD. Brown-Forsythe and Welch ANOVA followed by Dunnett’s multiple comparison tests. (**b**) Western-blot analysis of the relative protein expression of MCT1 in brain microvessels. For quantitative comparison, signals were normalized upon the signal from the endothelial-specific marker CD31. Mean ± SD. One-way ANOVA with Holm-Sidak’s multiple comparisons test. (**c**) Uptake clearance of [^14^C]-Lactate measured by brain in situ perfusion. Mean ± SD. One-way ANOVA with Holm-Sidak’s multiple comparisons test

The above results led us to hypothesize that the activation of PPAR δ might be responsible for the induction of *Slc16a1*/MCT1 in CECs during fasting. To directly test this hypothesis, rats were dosed with the specific PPAR δ antagonist GSK3787 (2 mg/kg i.p. every 12 h) while fasting. This antagonist was preferred over the one used in vitro (GSK0660) because it shows a more favorable bioavailability due to higher plasma maximal concentration and half-time in vivo [35]. For this experiment, rats were fasted for two days because the gene induction of *Slc16a1* was maximal at this time point. The gene expression of *Angptl4* was increased by seven-fold in the soleus muscle of the fasted rat as compared to fed rats and this increase was partially reversed by GSK3787 treatment, showing that we could effectively inhibit PPAR δ in vivo, at least partly (Fig. 7a). As shown in Fig. 7b, the protein expression of MCT1 doubled in the brain microvessels of rats fasted for two days as compared to rats fed *ad libitum* and GSK3787 administration almost completely blocked this induction. Morever, GSK3787 also reduced the basal expression of MCT1 in fed rats, suggesting that the PPAR δ signalization pathway is not only activated by fasting but also basally active. Finally, we asked whether the fasting-induced increase of MCT1 in the brain endothelium was accompanied by an increase in the uptake of its substrates at the BBB by measuring the uptake clearance (K_in_) of [^14^C]-L-Lactate using in situ brain perfusion. As shown in Fig. 7c, fasting significantly increased the K_in_ of [^14^C]-L-Lactate by 23%. In line with MCT1 expression results obtained by western-blot, the K_in_ value was decreased by GSK3787 administration although without reaching statistical significance between the F2 and F2 + GSK groups.

Altogether, the results obtained both in vitro and in vivo strongly suggest that the increase in the expression of *Slc16a1*/MCT1 in rat brain endothelial cells during fasting is the consequence of PPAR δ activation in these cells.

## Discussion

To our knowledge, the present study is the first to examine the global effect of fasting upon the brain endothelial cells which support the BBB. Its main result is the demonstration that fasting triggers an adaptive response of the brain endothelium involving PPAR δ activation and upregulation of MCT1.

Importantly, the rats adapted to fasting in a way similar to the human including a decrease in body weight, lowered glycemia, higher plasma concentration of free fatty acids and increased ketonemia, without showing any sign of muscle catabolism or denutrition. Moreover, the change in body weight after a three days fasting (-10.7%) was similar to the one observed in the healthy non-obese human following a 10 days fasting (-9.8%), a result consistent with the fact that rodents have a 4–7 fold higher basal metabolic rate per kg than human [36]. This suggests that our animal model and experimental design are well suited to explore the adaptation mechanisms that take place in the human subjected to a 1–10 days therapeutic fasting although interspecies differences are likely to exist.

Information about the expression and physiological function of PPARs at the BBB, and in particular in CECs, is limited. Several studies have reported PPAR α activation by pharmacological agonists such as fibrates in CECs and rodent brain microvessels both in vitro and in vivo [12, 37–39]. PPAR γ activation in CECs has been reported to dampen inflammation [40–45], although it should be noticed that most of these studies used PPAR γ agonists, such as rosiglitazone and pioglitazone, at concentrations which might also activate other PPAR isoforms [46]. PPAR δ has been established as a key nuclear receptor for peripheral vascular physiology through its action on endothelial and vascular smooth muscle cells [47]. In the brain, it is broadly expressed in various cell types [31, 48, 49] and it can dampen oxidative stress and neuroinflammation [50]. However, the contribution of the brain endothelium to these effects is poorly documented. By inhibiting the expression of miR-15a, endothelial PPAR δ mitigates apoptosis and cerebrovascular dysfunction following in vitro oxygen-glucose deprivation. Accordingly, intra-cerebroventricular infusion of GW501516 in mice subjected to ischemic brain injury reduced caspase-3 activation and DNA fragmentation in isolated brain microvessels and decreased the infarct size and extent of BBB disruption [51]. In another study, prostacyclin and its clinically approved analog iloprost activated PPAR δ in CECs, resulting in the augmented production of the neuroprotective soluble amyloid precursor protein sAPPα [52]. Of note, all these studies used pharmacological PPAR activation and whether these nuclear receptors play a role in the cerebrovascular system in physiological situations such as fasting was not addressed.

Our transcriptomic profiling of freshly isolated CECs from fed vs. fasted rats revealed that *Ppard* and several typical PPAR target genes (such as *Angptl4*, *Cpt1a*, *Hmgcs2, Mlycd*, *Pdk4*) are upregulated during fasting, while the expression of *Ppara* and *Pparg* is left unchanged, suggesting that PPAR δ is selectively activated in rat CECs during fasting. Moreover, in agreement with several transcriptomic studies using RT-PCR or bulk and single cell-RNA-Seq in the rodent brain [31, 48, 49, 53], *Ppard* is by far the most highly expressed isoform in CECs relative to *Ppara* and *Pparg*, while *Ppard* transcripts are highly enriched in purified CECs relative to whole cortex. Together, these data point to a specific function of PPAR δ in the cerebrovascular system, which was confirmed in vitro using pc-CECs.

In pc-CECs, PPAR δ specific pharmacological agonists strongly induced typical PPAR target genes (*Angptl4*, *Cpt1a*), whereas specific agonists of PPAR α and PPAR γ were ineffective. Moreover, the natural PPAR ligands palmitate and oleate, whose concentration rises in the plasma during fasting, also increased *Angptl4* and *Cpt1a* mRNA expression and their effects were completely blocked by GSK0660 (a specific PPAR δ antagonist) but not GW6471 (a specific PPAR α antagonist). Control experiments with the widely used PPAR γ antagonist GW9662 were not performed because this synthetic compound is known to have off-targets effects, including antagonist or agonist activity upon PPAR δ [54]. However, *Pparg* transcripts are detected at such a low level in brain endothelial cells that the transcriptional effects of fatty acids are unlikely to be driven by PPAR γ activation, as confirmed by the absence of detectable effect of rosiglitazone.

Interestingly, these results are similar with the ones obtained in the skeletal muscle where PPAR δ is also the most highly expressed isoform. In addition, while the expression of target genes such as *Angptl4* and *Cpt1a* are under the control of PPAR α in the liver, they are also exclusively regulated by PPAR δ in the muscle [55, 56]. In this organ, PPAR δ is pivotal in the metabolic adaptation to physiological situations such as fasting and exercise, in particular by stimulating fatty acids uptake and catabolism, while inhibiting glucose utilization [16]. In the present study, using a non-targeted proteomic approach, we demonstrate that PPAR δ activation selectively upregulates 60 proteins in primary cultured rat CECs, including several enzymes involved in the catabolism of lipids such as ACADL, ACADS, CPT1A, CPT2, MGLL and LIPS. Moreover, the expression of these six proteins was also found upregulated at the transcript level in rat brain CECs following fasting. Although it remains to be thoroughly investigated, it is tempting to speculate that, during fasting, cerebral endothelial cells increase their use of fatty acids as an energy nutrient, possibly to spare glucose for the brain parenchyma.

None of the PPAR target genes *Angptl4*, *Cpt1a*, and *Hmgcs2* (not shown) was upregulated by 20 µM fenofibrate in primary cultures of rat CECs. This does not rule out the possibility that higher concentration of fenofibrate or other PPAR α pharmacological agonists might be effective, as previously reported in brain endothelial cells and rodent brain microvessels both in vitro and in vivo [12, 37–39]. However, our results suggest that, relative to PPAR δ, PPAR α likely plays a minor role in the brain endothelium in physiological situations, as demonstrated here in fasting. As previously reported [31, 48, 49, 53], we confirm here that *Pparg* transcripts are barely detectable in brain endothelial cells and that PPAR target genes are not induced by rosiglitazone at pharmacologically active concentration (1 µM). This is in striking contrast with some studies which reported that PPAR γ activation might have anti-inflammatory action in brain endothelial cells [40–45]. Of note, very high concentrations (5–10 µM) of PPAR γ agonists, such as rosiglitazone and pioglitazone, were often used in these studies [40–43], although such concentrations are known to also engage PPAR α and PPAR δ [46]. It is thus difficult to unambiguously ascribe the observed effects to specific PPAR γ activation. However, since most of these studies have examined PPAR γ signaling in the context of inflammation [40, 41, 43–45], it remains possible that PPAR γ, while not expressed in quiescent or unstimulated endothelial cells, becomes upregulated in inflamed cells. This deserves further investigation.

The transporter of ketone bodies *Slc16a1*/MCT1 plays an essential role at the BBB as exemplified by the severe neurological picture observed in human patients suffering from inherited MCT1 deficiency [57]. When plasma glucose is low, as in the neonatal and suckling periods, during fasting, or when the diet is very low in carbohydrates (ketogenic diet), MCT1 fulfills an essential function by allowing alternate energy nutrients, such as lactate and ketones, to enter the brain and sustain neuronal activity [13, 58, 59]. In addition, MCT1 is able to facilitate the cerebral entry of several drugs including valproate, salicylate or the drug candidate 3-bromopyruvate [60, 61]. Several transcription factors, including PPAR α, are known to participate in the regulation of MCT1 in peripheral organs, such as the liver [33, 62, 63]. In the rat brain endothelial cell line RBE4, exposure to cAMP or activation of the canonical Wnt/b-catenin pathway increase MCT1 expression and activity by modulating its intracellular trafficking [64–66]. However, whether other signaling pathways are involved in MCT1 regulation at the BBB remained unknown.

Our present findings show that PPAR δ activation is responsible for the upregulation of *Slc16a1*/MCT1 in CECs of the fasted rat. First, physiological concentration of palmitate and oleate, the two most abundant FFAs in the plasma, increased MCT1 at the transcript and protein level in primary cultured rat CECs and this induction was reversed by PPAR δ, but not PPAR α, selective antagonists. Second, the gene expression of *Slc16a1* was unchanged following exposure to PPAR α and PPAR γ agonists but increased upon treatment with the PPAR δ agonist GW0742, an effect which was blocked by the specific PPAR δ antagonist GSK0660. Moreover GW0742 also upregulated MCT1 expression in a GSK0660-sensitive manner as demonstrated by non-targeted proteomics. Finally, according to our in vitro findings, MCT1 was upregulated in the brain microvessels of fasted rats and this effect was completely reversed when rats were dosed with the specific PPAR δ antagonist GSK3787 while fasting. MCT1 upregulation in the fasted rats was also associated with an increase in the brain uptake clearance of [^14^C]-L-Lactate and this increase was reduced by treatment with the PPAR δ specific antagonist GSK3787 (although without reaching statistical significance). Of note, the observed increase in the K_in_ value for [^14^C]-L-Lactate, albeit significant, was rather small (+ 27%) as compared to the almost doubling (+ 101%) in MCT1 protein expression. One hypothesis to explain such a difference might be that only a fraction of the upregulated transporter is localized and functional at the luminal plasma membrane. Another possibility is that the lactate uptake clearance might be decreased by an outward flux from the highly glycolytic endothelial cells. Altogether these results confirm that the fasting-induced upregulation of MCT1 in the rat cerebrovascular system is mediated by PPAR δ.

Although the above results strongly support that *Slc16a1* is under the transcriptional control of PPAR δ in rat CECs, we observed that, contrary to GW0742 and free fatty acids, the other specific PPAR δ synthetic agonist GW501516 was unable to upregulate the gene expression of *Slc16a1 in vitro*. We hypothesize that these seemingly discrepant results might be related to the fact that distinct ligands can trigger distinct conformational changes of PPAR δ and thus binding of distinct cofactors, resulting in complexes with distinct transcriptional activities. Although it remains to be studied in details for PPAR δ, this concept has been demonstrated for PPAR α and PPAR γ and underlies the development of promising selective peroxisome proliferator-activated receptor modulators (SPPARMs) [67, 68].

There is only one report of the effect of fasting upon MCT1 expression in brain cortex microvessels [13]. In this study, no change in the expression of MCT1 could be detected by western-blot which stands in contradiction with our results. We have no definite explanation for this discrepancy but it might stem from differences in the metabolic switch in the experimental animals used. In particular, although we both report a drop in glycemia and a rise in ketonemia after a 48 h fasting, the plasma FFAs concentration did not change in the above-cited study (performed in male Fisher rats) while it significantly doubled from 0.3 ± 0.1 mM to 0.6 ± 0.2 mM in the present study (performed in male Wistar-Han rats). This might be relevant in light of the role that circulating FFAs might play in the induction of MCT1 through PPAR δ activation. Moreover a rise in plasma FFAs concentration is a characteristic feature of the physiological adaptation of healthy humans to fasting [69, 70] suggesting that Wistar-Han rats might be a better animal model than Fisher rats in these experiments.

Besides our findings that fasting upregulates MCT1 via PPAR δ activation in CECs, the present study also yields unprecedented information regarding the impact of fasting on the transcriptional profile of brain endothelial cells, in particular their specific repertoire of ABC and SLC transporters, a hallmark of the BBB.

The blood-to-brain transport of most drugs is highly dependent on the endothelial expression and activity of ABC transporters, which mediate their unidirectional efflux, and SLC transporters, which can facilitate bidirectional transport [71]. ABC transporters can accommodate a large range of structurally unrelated compounds and thus strictly limit the brain uptake of a wide variety of drugs. On the contrary, SLC transporters are often highly selective and thus can mediate the brain uptake of selected drugs, with the exception of the transporters belonging to the *Slc22* (OATs/OCTNs) and *Slco* (OATPs) families, which can handle a larger panel of drugs. The expression of the main drug efflux ABC transporters at the BBB, including PGP, BCRP and MRP4, was not changed at the transcript level or protein level following one to three days of fasting. Moreover, the activity of the major transporter PGP, as measured by in situ brain perfusion, was not modified by fasting. Of note, it was previously reported that one day of fasting increased PGP activity at the mouse BBB [12] but this study assessed PGP activity ex vivo in isolated brain microvessels and did not measure PGP expression in the microvessels. BCRP is also able to limit the brain penetration of a large array of drugs, most of which are shared substrates with PGP [72]. In mouse and rat brain microvessels, PGP expression is about 4 times higher than BCRP expression [73, 74] and consequently BCRP plays a less significant role at the rodent BBB, except for the few drugs that are selective substrates of BCRP [75]. This might be different in the human and non-human primates where BCRP and PGP are expressed at a similar level at the BBB [76, 77], although the relative contribution of each transporter in limiting the drug penetration in the brain remains to be clearly established [72]. Therefore, from a translational perspective, further studies should address the impact of fasting upon BCRP function in more relevant preclinical models such as the non-human primate. Our transcriptomic study also showed that fasting modulated the gene expression of some SLC transporters involved in drug transport, including *Slc19a3*/THTR2 (metformin), *Slc38a5*/SNAT5 (platinum) and *Slc16a1*/MCT1 (valproate) as well as *Slco1a4* and *Slco1c1* but not the major drug handling transporters of the *Slc22* family. Altogether these results indicate that, except for a restricted set of compounds, fasting should not significantly alter the cerebral pharmacokinetic profile of most drugs, at least in the male rat and in the investigated time window (one to three days).

Because they control the cerebral uptake of most endogenous compounds and metabolites, SLC transporters are a major actor in brain homeostasis. Unsurprisingly, the gene expression of the transporters of glucose (*Slc2a1*/GLUT1) and ketone bodies (*Slc16a1*/MCT1), the two main energy substrates of the brain, was upregulated following fasting [9]. The transporter of glutamine (*Slc38a3*/SNAT3), known to be highly expressed in the brain endothelium [78] was also induced and this might be functionally relevant given the role of glutamine for energy supply, nitric oxide synthesis and glutamate and GABA homeostasis in the brain. Interestingly two transporters of neurotransmitters including glutamate (*Slc1a1*/EAAT3) and taurine (*Slc6a6*/TAUT) were, respectively, up- and down-regulated. In the mouse brain, these transporters are highly expressed in endothelial cells [31, 48, 49], where they take part in the clearance of the pro-convulsive glutamate [79] and anti-convulsive taurine [80]. Whether the fasting-induced changes observed in the expression of *Slc1a1* and *Slc6a6* in the brain endothelium participate in the anti-convulsive effect of fasting is an attractive hypothesis. Finally, the induction of the transporters of the essential enzymatic cofactors thiamine (*Slc19a3*/THTR2) and riboflavine (*Slc52a2*/RFT3) suggest that brain endothelial cells actively participate in the metabolic adaptation of the brain to low-energy stress. Taken together, these results suggest that modulation of the BBB by fasting might be a valuable approach to tackle metabolic dysfunction of the brain, although this remains to be specifically addressed.

### Limitations of the study

This work was conducted only in male rats and its results cannot be generalized to female animals without additional studies. The effect of refeeding and the long-term effect of repeated bouts of fasting upon the brain endothelium were beyond the scope of this study but clearly deserves further investigation.

## Conclusions

In conclusion, the present study reveals that fasting, a mild metabolic stressor, triggers an adaptive response of the cerebrovascular system through selective PPAR δ activation in endothelial cells. This in turn upregulates MCT1 expression and activity, thereby contributing to the sustained energy supply of the brain during fasting. Considering the established protective role of PPAR δ in the peripheral vasculature, our results should motivate further investigation of fasting as a way to modulate BBB function and target brain diseases associated to metabolic dysfunction.

### Electronic supplementary material

Below is the link to the electronic supplementary material.

**Supplementary Figure 1** Metabolic effect of fasting. Rats were fed *ad libitum* (AL)or fasted for one day (F1), two days (F2) or three days (F3). (**a**) Plasma Total proteins and Urea. (**b**) Relative weights (% of body weight) of the heart, gastrocnemius, extensor digitorum longus (EDL) and soleus muscles. Tukey boxplot (n = 12 rats per group). Kruskal-Wallis test with Dunn’s multiple comparisons test for Total Proteins. Brown-Forsythe and Welch ANOVA followed by Dunnett’s multiple comparison tests for Gastrocnemius. One-way ANOVA followed by Holm-Sidak’s multiple comparisons test for all other variables.

**Supplementary Table S1** Transcriptomic dataset of endothelial cells freshly isolated from the brain of rats fed ad libitum (AL) or fasted for 1, 2, or 3 days (F1, F2, or F3).

**Supplementary Table S2** Differentially expressed ABC and SLC transporters encoding genes. Data extracted from the whole transcriptomic dataset of endothelial cells freshly isolated from the brain of rats fed ad libitum (AL) or fasted for 1, 2, or 3 days (F1, F2, or F3).

**Supplementary Table S3** Proteomic dataset of primary cultured rat brain endothelial cells treated with 100 nM GW0742, 100 nM GW0742 + 2 µM GSK0660 or left untretated (n=3).

**Supplementary Table S4** Primary and secondary antibodies for western-blot

**Supplementary Table S5** Primers used for qRT-PCR (5’ to 3’ sequence)

## Data Availability

Data are available on request from the corresponding author. The RNASeq gene expression data and raw fastq files are available on the GEO repository (https://www.ncbi.nlm.nih.gov/geo/) under accession number GSE241803. The proteomics raw and result files have been deposited to the ProteomeXchange Consortium (http://proteomecentral.proteomexchange.org) via the jPOSTrepo partner repository with the dataset identifiers PXD044587 and JPST002293, respectively.

## Abbreviations

ABC transporter: ATP-Binding Cassette transporter
AL: *Ad Libitum*
BBB: Blood-Brain Barrier
CECs: Cerebral Endothelial Cells
FFA: Free Fatty Acid
MACS: Magnetic-activated cell sorting
pc-CECs: primary cultured Cerebral Endothelial Cells
SLC transporter: Solute Carrier transporter
