# Supplementary material for: Fasting upregulates the monocarboxylate transporter MCT1 at the rat blood-brain barrier through PPAR δ activation
Source: Fluids Barriers CNS. 2024 Apr 8;21:33. doi: 10.1186/s12987-024-00526-8 (PMC11003008; doi:10.1186/s12987-024-00526-8)
Supplement: Supplementary file 1 — Supplementary Figure 1 Metabolic effect of fasting. Rats were fed ad libitum (AL)or fasted for one day (F1), two days (F2) or three days (F3). (a) Plasma Total proteins and Urea. (b) Relative weights (% of body weight) of the heart, gastrocnemius, extensor digitorum longus (EDL) and soleus muscles. Tukey boxplot (n = 12 rats per group). Kruskal-Wallis test with Dunn’s multiple comparisons test for Total Proteins. Brown-Forsythe and Welch ANOVA followed by Dunnett’s multiple comparison tests for Gastrocnemius. One-way ANOVA followed by Holm-Sidak’s multiple comparisons test for all other variables. [file 12987_2024_526_MOESM1_ESM.docx]

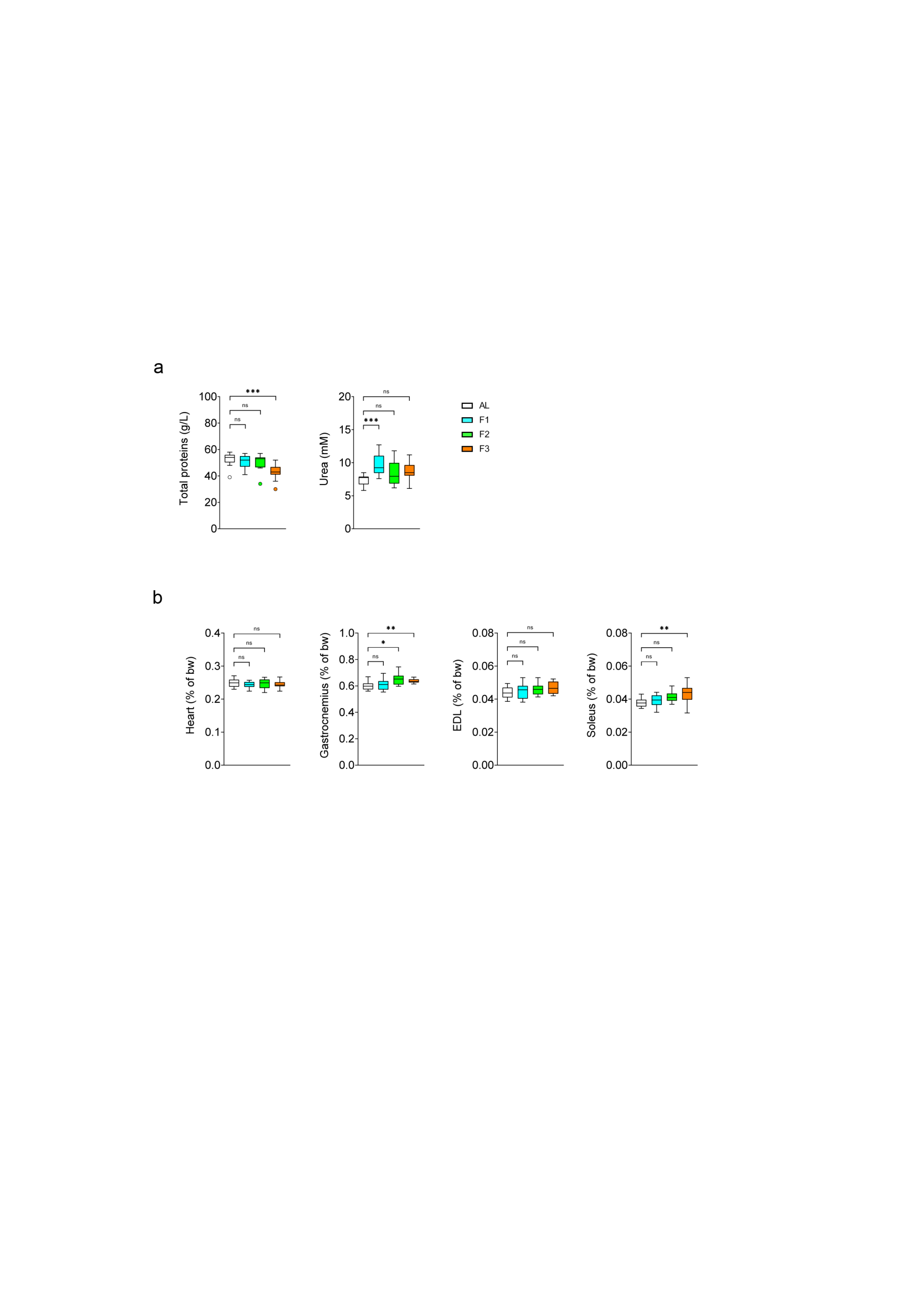


**Supplementary Figure 1** Metabolic effect of fasting. Rats were fed *ad libitum* (AL) or fasted for one day (F1), two days (F2) or three days (F3). (**a**) Plasma Total proteins and Urea. (**b**) Relative weights (% of body weight) of the heart, gastrocnemius, extensor digitorum longus (EDL) and soleus muscles. Tukey boxplot (n = 12 rats per group). Kruskal-Wallis test with Dunn’s multiple comparisons test for Total Proteins. Brown-Forsythe and Welch ANOVA followed by Dunnett’s multiple comparison tests for Gastrocnemius. One-way ANOVA followed by Holm-Sidak’s multiple comparisons test for all other variables.

**
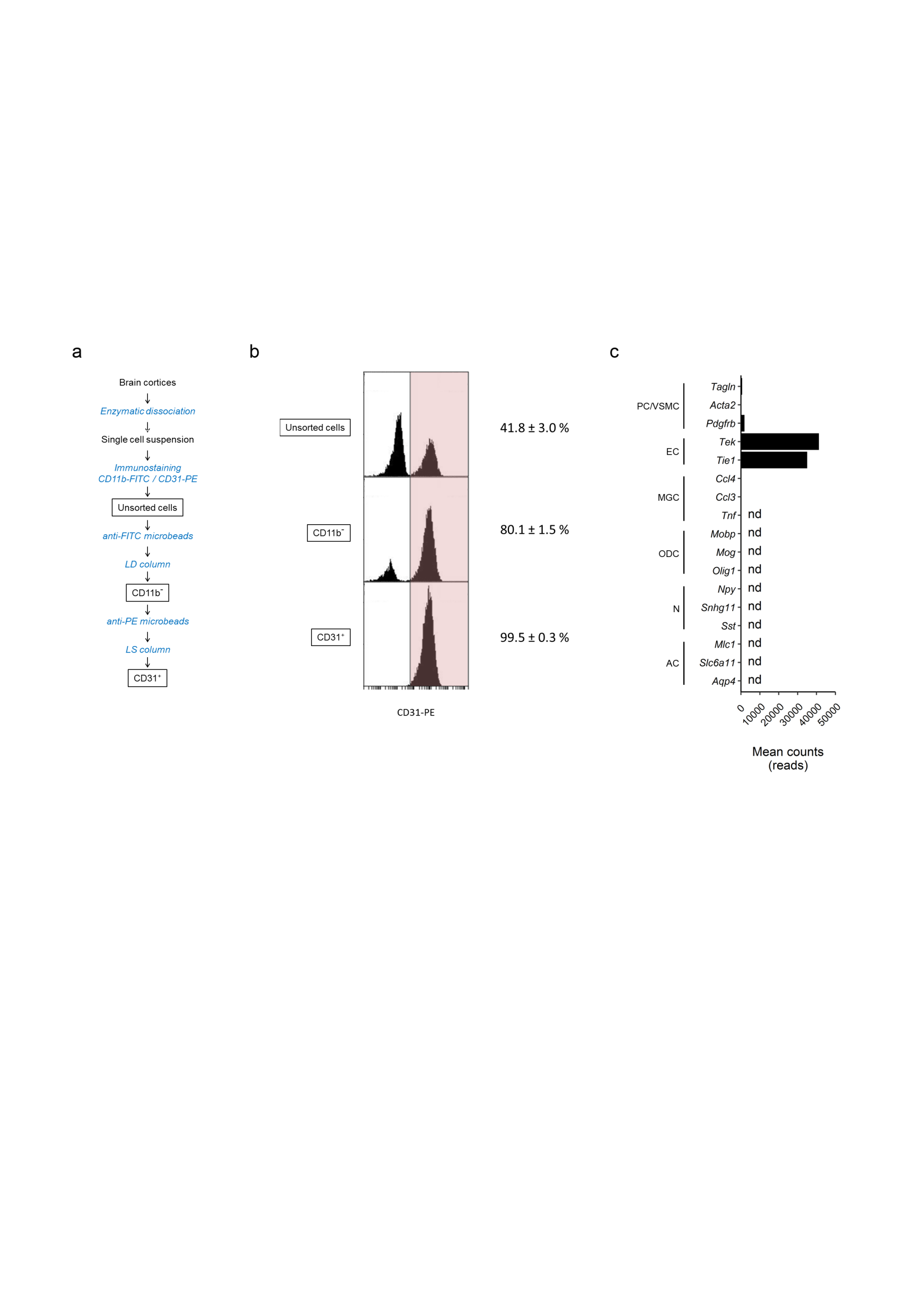
**

**Supplementary Figure 2** Preparation and characterization of rat cerebral endothelial cells freshly isolated by magnetic-assisted cell sorting (MACS). (**a**) Workflow of the whole procedure (see Methods for details). (**b**) PE (phycoerythrin) fluorescence distribution of the unsorted cells, CD11b^-^ cells, and CD31^+^ cells as assessed by flow cytometry. The percentage of CD31^+^ endothelial cells is shown on the right. (**c**) Mean counts of mural cells (PC/VSMC), endothelial cells (EC), microglia (MGC), oligodendrocytes (ODC), neurons (N) and astrocytes (AC) specific transcripts as measured by RNA-Seq (nd = not detected).
