# Supplementary material for: Fasting upregulates the monocarboxylate transporter MCT1 at the rat blood-brain barrier through PPAR δ activation
Source: Fluids Barriers CNS. 2024 Apr 8;21:33. doi: 10.1186/s12987-024-00526-8 (PMC11003008; doi:10.1186/s12987-024-00526-8)
Supplement: Supplementary file 4 — Supplementary Table S3 Proteomic dataset of primary cultured rat brain endothelial cells treated with 100 nM GW0742, 100 nM GW0742 + 2 µM GSK0660 or left untretated (n=3). [file 12987_2024_526_MOESM4_ESM.pdf]

Supplementary Table S4. Primary and secondary antibodies for western-blot

**Antibody**	**Manufacturer**	**Reference**	**Lot number**	**Dilution**
mouse anti-Pgp	Enzo Life Sciences	ALX-801-002	08181607	1/100
rat anti-Bcrp	Enzo Life Sciences	ALX-801-036	05062127	1/50
goat anti-Mrp4	Abcam	ab77184	GR54544-2	1/500
goat anti-CD31	R&D Systems	AF3628-SP	YZU0121082	1/500
chicken anti-Mct1	Merck	AB1286-I	3674956	1/2000
rabbit anti-βActin	Abcam	Ab49900	GR3324650-1	1/50000
Anti-Mouse HRP	Invitrogen	A16011	67-51-041519	1/20000
Anti-Rabbit HRP	Invitrogen	A16023	65-122-031219	1/20000
Anti-Goat HRP	Invitrogen	PA1-28664	WB3206854P	1/5000
Anti-Chicken HRP	Invitrogen	SA1-72004	VL3141722	1/15000
