# Supplementary material for: Fasting upregulates the monocarboxylate transporter MCT1 at the rat blood-brain barrier through PPAR δ activation
Source: Fluids Barriers CNS. 2024 Apr 8;21:33. doi: 10.1186/s12987-024-00526-8 (PMC11003008; doi:10.1186/s12987-024-00526-8)
Supplement: Supplementary file 5 — Supplementary Table S4 Primary and secondary antibodies for western-blot [file 12987_2024_526_MOESM5_ESM.pdf]

Supplementary Table S5. Primers used for qRT-PCR (5’ to 3’ sequence)

Gene	Primer sequence
Abcb1a	F : CAACCAGCATTCTCCATAATA<br>R : CCCAAGGATCAGGAACAATA
Ppara	F : CGCAGGAAAGACTAGCAACAATC<br>R : ACCTCTGCCTCCTTGTTTTCAA
Ppard	F : AGAGCACACCCTTCCTTCCA<br>R : CCCCATCACAGCCCATCT
Pparg	F : AGGGCGATCTTGACAGGAAAG<br>R : TGCAGGGGGGTGATATGTTTGA
Angptl4	F : AACGCCACCCGCTTACACA<br>R : AGAGGCTGGATCTGGAAAAGT
Cpt1a	F : CGAGTCCCGATGCCTTCA<br>R : CAGTCTCTGTCCTCCCTTCT
Slc16a1	F : AGTGCAACGACCAGTGAAGT<br>R : AGCCACCAGCAATCATTACT
Tbp	F : TGCACAGGAGCCAAGAGTGAA<br>R : CACATCACAGCTCCCCACCA
